# Hepatocellular Carcinoma and Non-Alcoholic Fatty Liver Disease: A Modern Context for an Ancient Disease

**DOI:** 10.3390/jcm12144605

**Published:** 2023-07-11

**Authors:** Paolo Gallo, Marianna Silletta, Federica Lo Prinzi, Tommaso Farolfi, Alessandro Coppola

**Affiliations:** 1 Operative Research Unit of Clinical Medicine and Hepatology, Fondazione Policlinico Universitario Campus Bio-Medico, Via Alvaro del Portillo, 200-00128 Rome, Italy; paolo.gallo@policlinicocampus.it; 2 Operative Research Unit of Oncology, Fondazione Policlinico Universitario Campus Bio-Medico, Via Alvaro del Portillo, 200-00128 Rome, Italy; m.silletta@policlinicocampus.it (M.S.); f.loprinzi@policlinicocampus.it (F.L.P.); 3 General Surgery Unit, Fondazione Policlinico Universitario Campus Bio-Medico, Via Alvaro del Portillo, 200-00128 Rome, Italy; 4 Dipartimento di Chirurgia, Sapienza University of Rome, Viale Regina Elena 291, 00161 Rome, Italy; coppola.chirurgia@gmail.com

Hepatocellular Carcinoma (HCC) is a leading cause of cancer-related deaths worldwide. Typically, HCC arises in a cirrhotic liver, with chronic liver diseases caused by hepatitis B virus (HBV) or hepatitis C virus (HCV) being the primary risk factors for several decades. Alcoholic liver disease is also a significant risk factor. In the case of chronic HBV infection, the development of HCC can occur even before cirrhosis manifests, with the estimated risk ranging from 10 to 25%. Chronic HCV infection, on the other hand, is associated with an increased risk of HCC primarily due to the development and progression of cirrhosis [1].

In addition to hepatotropic viral infections, cirrhosis is a major contributing factor to the development of HCC, as it represents a pre-neoplastic condition [2]. Non-alcoholic fatty liver disease (NAFLD) has emerged as the leading cause of chronic liver disease globally, encompassing a range of conditions from simple liver steatosis to inflammation and hepatocellular injury (non-alcoholic steatohepatitis, NASH), and ultimately progressing to fibrosis and cirrhosis [2]. Furthermore, NAFLD serves as the underlying cause for the rising incidence of HCC, with the annual occurrence of NAFLD-related HCC projected to increase by 45–130% by 2030 [3].

The highest risk of HCC is observed in patients with advanced fibrosis or cirrhosis. However, a significant number of studies have demonstrated that 20–50% of HCC cases occur in individuals with NAFLD, even in the absence of cirrhosis [4,5]. Recently, a new term, “metabolic-associated-fatty-liver-disease” (MAFLD), has been introduced in the literature to highlight the coexistence of steatosis and metabolic dysfunctions [6]. The pathogenesis of NAFLD is strongly linked to metabolic dysfunctions, and it is important to note that NAFLD is a term that indicates the absence of alcohol-related liver disease. Insulin resistance, hyperinsulinemia, oxidative stress, activation of hepatic stellate cells, cytokine/adipocytokine signaling pathways, and genetic and environmental factors are all key factors in the development and progression of NAFLD-related HCC. The risk factors and underlying mechanisms for HCC development are multifactorial and not fully understood [7].

Nevertheless, the prevailing hypothesis suggests that insulin resistance, obesity, and inflammation directly contribute to carcinogenesis, particularly in patients without cirrhosis. This process involves oxidative stress, protein damage, and innate immune system activation, which collectively induce DNA damage and create a favorable microenvironment for HCC [8]. Additionally, accumulating evidence highlights the role of genetics in the interindividual variability across all spectra of NAFLD, including the development of HCC [9,10], even in patients without cirrhosis. Among various genetic factors, patatin-like phospholipase-3 (PNPLA3) has been identified as an independent risk factor for HCC, particularly in individuals with non-alcoholic steatohepatitis or alcoholic liver disease (odds ratio 1.67) [11].

The current management of patients with “metabolic” HCC does not differ from other etiologies. However, the new epidemiological landscape has presented us with patients with better liver function and no decompensated cirrhosis [12]. Nevertheless, these patients often exhibit a more complex clinical picture due to metabolic and cardiovascular comorbidities. In terms of therapy, significant advancements have improved the management of patients with advanced HCC. Notably, the distinct pathogenesis of NAFLD-HCC compared to viral HCC has garnered considerable attention.

Since the introduction of Sorafenib [13], the first tyrosine kinase inhibitor (TKI) to demonstrate a benefit in overall survival (OS) in a phase III trial for advanced HCC in 2008, several other drugs have been tested as first-line treatments. However, most failed to show superiority over placebo due to toxicity or inefficacy. In 2018, lenvatinib [14], another multi-TKI, was found to be non-inferior to Sorafenib in terms of OS, with a potential advantage in progression-free survival (PFS) and a notable response rate.

Subsequently, immune checkpoint inhibitors (anti-PD1, anti-PDL1, and anti-CTLA-4) have been evaluated as monotherapy in the second line for patients with advanced HCC, with response rates ranging from 15 to 20%. However, no survival benefits were observed until the combination of atezolizumab plus bevacizumab (AB) [15] demonstrated superiority over Sorafenib in a first-line phase III trial, with a median OS of 19.2 months (95% CI 17.0–23.7) for AB and 13.4 months (95% CI 11.4–16.9) for Sorafenib (hazard ratio [HR] 0.66; 95% CI 0.52–0.85; descriptive *p* < 0.001). The AB combination also showed significantly improved PFS and response rate, establishing it as the standard of care worldwide for first-line treatment.

Despite efforts to identify predictive and prognostic biomarkers, none of the known markers have proven useful in determining the best systemic strategy and treatment choice for patients with advanced disease. Consequently, with the new epidemiological and pathophysiological understanding, attention has shifted toward the role of liver disease etiology in determining the response to systemic treatment. Retrospective analyses were conducted following interesting preclinical data by Pfister et al. [16], which suggested that mice with non-viral HCC, particularly NASH-HCC, were less responsive to immunotherapy.

In a retrospective analysis of 1341 patients treated in the first line with AB or lenvatinib [17], after balancing patient characteristics using inverse probability of treatment weighting (IPTW) methodology, OS was significantly improved in the NAFLD/NASH subgroup treated with lenvatinib (HR: 1.88; 95% CI: 1.16, 3.01; *p* = 0.014). In the viral subgroup, patients treated with AB had superior OS (HR: 0.76; 95% CI: 0.61, 0.96; *p* = 0.024). These retrospective findings require prospective validation. Until confirmed, the etiology of liver disease cannot be considered a factor influencing the treatment algorithm.

Finally, second-line and subsequent lines of systemic treatment have been registered and approved. However, the optimal drug sequences have yet to be clarified due to a lack of helpful data in the literature regarding strategies after progression from first-line treatment. Currently, numerous clinical trials are investigating the efficacy of new combinations of immune checkpoint inhibitors not only in the second line but also in early-phase treatment.

Surgical management for NAFLD-HCC does not differ from other etiologies of HCC, and therapeutic options include hepatic resection and liver transplantation. However, the impact of surgical resection on overall survival in metabolic HCC remains unclear. Several series of patients with metabolic-related HCC who underwent surgical resection have been investigated to compare short- and long-term outcomes with other etiologies of HCC. However, due to variations in variable definitions and study designs, no consensus has been reached.

In a retrospective analysis by Pal Chaudhary et al., a series of 260 resected patients with different etiologies of HCC were analyzed. Their results showed that NAFLD/NASH-related HCC had a lower fibrosis grade in the final pathological report compared to viral etiologies but had a higher median tumor size. Apart from these two characteristics, no other differences were observed regarding other high-risk features or overall survival [18].

Conci et al., in a study including over 1000 resected patients, found that patients with metabolic HCC had an increased rate of cardiovascular and infectious complications after surgery. Due to the higher rate of comorbidities, their post-operative course was associated with an increased operative risk and a doubled post-operative mortality rate. Furthermore, NAFLD-HCC had significantly worse survival than other groups, serving as an independent prognostic factor for overall survival [19]. These findings were partially confirmed by a meta-analysis conducted by Su in 2023, which analyzed over 11,477 cases. Contrary to previous reports, this study showed comparable rates of post-operative complications between the two patient groups. Additionally, no differences in overall survival and disease-free survival were found after surgery. Interestingly, when limited to the Asian population, patients with metabolic etiology of HCC had a better prognosis in terms of overall survival and disease-free survival [20].

These data highlight the lack of clear evidence regarding surgical treatment in patients with metabolic HCC. Similar to viral HCC, liver transplantation remains a potentially curative approach for localized disease in these patients, and NAFLD-HCC is gaining importance as an indication for liver transplantation in the United States [18].

Recent publications by Rajendran et al. reported outcomes of liver transplantation in NAFLD-HCC. Approximately 10% of NAFLD-HCC cases underwent liver transplantation, with post-transplantation survival rates of 90.8%, 83.9%, and 76.3% at 1-, 3-, and 5-year intervals, respectively. No differences were observed in terms of cardiovascular-related death or HCC recurrence-related death. Overall survival was comparable between the two groups, justifying equivalent organ allocation despite the NAFLD status [21].

In conclusion, from a surgical perspective, there is currently no evidence of treatment differences among different etiologies of HCC. The published studies reveal the significant complexity of these patients, highlighting the crucial role of multidisciplinary management in optimizing patient outcomes, similar to patients with complex colorectal liver metastasis [22].

In this context, HCC represents a modern challenge, and further efforts are needed to better understand its clinical and therapeutic implications.
